# Prognostic Significance of Serum Albumin Level and Albumin-Based Mono- and Combination Biomarkers in Patients with Hepatocellular Carcinoma

**DOI:** 10.3390/cancers15041005

**Published:** 2023-02-04

**Authors:** Long-Bin Jeng, Wen-Ling Chan, Chiao-Fang Teng

**Affiliations:** 1 Organ Transplantation Center, China Medical University Hospital, Taichung 404, Taiwan; 2 Department of Surgery, China Medical University Hospital, Taichung 404, Taiwan; 3 Cell Therapy Center, China Medical University Hospital, Taichung 404, Taiwan; 4 Department of Bioinformatics and Medical Engineering, Asia University, Taichung 413, Taiwan; 5 Epigenome Research Center, China Medical University Hospital, Taichung 404, Taiwan; 6 Graduate Institute of Biomedical Sciences, China Medical University, Taichung 404, Taiwan; 7 Program for Cancer Biology and Drug Development, China Medical University, Taichung 404, Taiwan; 8 Research Center for Cancer Biology, China Medical University, Taichung 404, Taiwan

**Keywords:** hepatocellular carcinoma, albumin, prognostic significance, mono-biomarker, combination biomarker

## Abstract

**Simple Summary:**

This review provides a comprehensive summary of the literature published thus far to highlight the prognostic significance of serum albumin (ALB) level and various ALB-based mono- and combination biomarkers in predicting the prognosis of patients with hepatocellular carcinoma (HCC) treated with different therapies.

**Abstract:**

Hepatocellular carcinoma (HCC) is the predominant form of primary liver cancer. Although many surgical and nonsurgical therapeutic options have been established for treating HCC, the overall prognosis for HCC patients receiving different treatment modalities remains inadequate, which causes HCC to remain among the most life-threatening human cancers worldwide. Therefore, it is vitally important and urgently needed to develop valuable and independent prognostic biomarkers for the early prediction of poor prognosis in HCC patients, allowing more time for more timely and appropriate treatment to improve the survival of patients. As the most abundant protein in plasma, human serum albumin (ALB) is predominantly expressed by the liver and exhibits a wide variety of essential biological functions. It has been well recognized that serum ALB level is a significant independent biomarker for a broad spectrum of human diseases including cancer. Moreover, ALB has been commonly used as a potent biomaterial and therapeutic agent in clinical settings for the treatment of various human diseases. This review provides a comprehensive summary of the evidence from the up-to-date published literature to underscore the prognostic significance of serum ALB level and various ALB-based mono- and combination biomarkers in the prediction of the prognosis of HCC patients after treatment with different surgical, locoregional, and systemic therapies.

## 1. Introduction

Hepatocellular carcinoma (HCC) is the most prevalent type of primary liver cancer and is responsible for up to 90% of primary liver malignancies [1,2]. Although remarkable progress has been made in the prevention and treatment of HCC, HCC remains the sixth most frequent human cancer and the third leading cause of cancer-related death worldwide, leading to nearly 900,000 new cases and 800,000 new deaths each year [3,4]. Although curative therapeutic options such as liver transplantation and hepatic resection surgery are available for the treatment of HCC patients, the survival benefits of the therapies are considerably impeded by donor-organ scarcity and recurrence of HCC after surgery, respectively [5,6]. Moreover, HCC displays a high degree of intratumoral heterogeneity and drug resistance, which largely weaken the therapeutic effectiveness of current locoregional and systemic treatments, such as radiation therapy, ablation, embolization, chemotherapy, and molecular targeted therapy [7,8,9]. Therefore, the discovery of valuable and independent prognostic biomarkers for the early identification of HCC patients with poor prognosis allowing for timely management and better treatment is an important objective to improve the survival of patients.

Human serum albumin (ALB) is the most abundant protein in plasma. ALB is prominently produced by and secreted from the liver and has multiple important biological functions, including regulation of plasma oncotic pressure, determination of tissue fluid distribution, maintenance of vascular permeability, adjustment of acid–base equilibrium, modulation of immune responses, suppression of oxidative stress, prevention of endothelial cell apoptosis, inhibition of blood coagulation, and transportation of endogenous and exogenous compounds [10,11]. In addition, it has been well verified that serum ALB level is a valuable and independent biomarker for a broad range of human diseases, including cancer, chronic liver disease, chronic kidney disease, ischemic stroke, diabetes, rheumatoid arthritis, inflammatory bowel disease, severe acute graft-versus-host disease, post-menopausal obesity, and the diseases that require glycemic control [12,13,14,15]. Moreover, ALB is widely used in clinical settings to treat a wide variety of human diseases, including chronic liver disease, acute liver failure, shock, trauma, burns, hypovolemia, hemorrhage, surgical blood loss, hemodialysis, cardiopulmonary bypass, resuscitation, nutritional support, acute respiratory distress syndrome, and hypoalbuminemia [16,17,18,19].

In this review, we comprehensively summarize the literature published thus far, providing supportive evidence to highlight the prognostic significance of serum ALB level and various ALB-based mono- and combination biomarkers in the prediction of the outcomes of HCC patients receiving different treatment modalities including surgical, locoregional, and systemic therapies.

## 2. Prognostic Significance of Serum ALB Level in HCC Patients

Multiple lines of studies have validated the prognostic value of a pre-treatment serum ALB level in HCC patients (Table 1). Studies conducted by Chen et al. [20], Chen et al. [21], and Wang et al. [22] showed that a low pre-treatment serum ALB level independently predicted worse overall survival (OS), disease-free survival (DFS), and recurrence-free survival (RFS) in HCC patients undergoing curative surgical resection. Another study conducted by Zeng et al. [23] revealed that a low pre-treatment serum ALB level was independently correlated with worse OS in HCC patients treated with external beam radiation therapy (EBRT). In addition, studies conducted by Castellano et al. [24] and Kuriyama et al. [25] identified that a low pre-treatment serum ALB level is a factor that is independently associated with worse OS in HCC patients treated with percutaneous ethanol injection (PEI). Studies conducted by Xu et al. [26] and Toshikuni et al. [27] demonstrated that a low pre-treatment serum ALB level independently predicted worse OS in HCC patients treated with radiofrequency ablation (RFA) or percutaneous microwave ablation (PMWA). Studies conducted by Ikeda et al. [28] and O’Suilleabhain et al. [29] confirmed that a low pre-treatment serum ALB level is an independent factor which predicts worse OS in HCC patients treated with transarterial embolization (TAE) or transarterial chemoembolization (TACE). Furthermore, a study conducted by Lerose et al. [30] ascertained that a low pre-treatment serum ALB level was independently associated with worse OS in HCC patients treated with curative surgical resection, PEI, TACE, or antihormonal therapy. Another study conducted by Baek et al. [31] verified that a low pre-treatment serum ALB level was an independent predictor for worse OS and failure-free survival (FFS) in HCC patients receiving molecular targeted therapy with sorafenib.

Collectively, these studies support the use of a low pre-treatment serum ALB level as a valuable independent prognostic biomarker for poor outcomes in HCC patients after treatment with various therapies.

## 3. Prognostic Significance of Serum ALB/Alkaline Phosphatase (ALP) Ratio in HCC Patients

Other than serum ALB level alone, many serum ALB-based biomarkers, which are developed based on ALB and various other factors, have been verified as having prognostic value in HCC patients in multiple lines of studies. One such biomarker is the ratio of the pre-treatment serum ALB level to the serum ALP level (Table 2). ALP is a kind of phosphatase that catalyzes the hydrolysis of various organic phosphate esters at alkaline pH values [32]. Studies conducted by Chan et al. [33], Li et al. [34], and Zhang et al. [35] showed that a low pre-treatment serum ALB/ALP ratio independently predicted worse OS, DFS, and RFS in HCC patients receiving curative surgical resection. Another study conducted by Li et al. [36] determined an independent association between a low pre-treatment serum ALB/ALP ratio and worse OS in HCC patients undergoing liver transplantation. Furthermore, a study conducted by Zhang et al. [37] confirmed that a low pre-treatment serum ALB/ALP ratio was independently correlated with worse OS and RFS in HCC patients treated with RFA. A study conducted by Chen et al. [38] identified a low pre-treatment serum ALB/ALP ratio as an independent factor which predicted worse OS in HCC patients treated with TACE. Another study conducted by Cai et al. [39] demonstrated that a low pre-treatment serum ALB/ALP ratio was a factor that was independently associated with worse OS in HCC patients treated without any standard anti-cancer therapies.

Taken together, these studies suggest that a low pre-treatment serum ALB/ALP ratio has a significant independent prognostic value for poor outcomes in HCC patients after treatment with various therapies.

## 4. Prognostic Significance of Serum C-Reactive Protein (CRP)/ALB Ratio in HCC Patients

The prognostic value of another serum ALB-based biomarker, which is the ratio of the pre-treatment serum CRP level to serum ALB level, in HCC patients has been validated in multiple lines of studies (Table 3). CRP is a plasma protein that is produced by the liver and functions as a key component in acute-phase inflammatory response [40]. Studies conducted by Shimizu et al. [41] and Ren et al. [42] showed that a high pre-treatment serum CRP/ALB ratio independently predicted worse OS and tumor-free survival (TFS) in HCC patients undergoing curative surgical resection. Studies conducted by Kinoshita et al. [43], Chen et al. [44], and Li et al. [45] revealed that a high pre-treatment serum CRP/ALB ratio was independently correlated with worse OS and RFS in HCC patients treated with curative surgical resection, RFA, PEI, or TACE. A study conducted by Tada et al. [46] ascertained an independent association between a high pre-treatment serum CRP/ALB ratio and worse OS in HCC patients receiving molecular targeted therapy with lenvatinib. Consistent with the aforementioned findings, a study conducted by Wu et al. [47] identified a low pre-treatment serum ALB/CRP ratio as an independent predictor for worse OS and DFS in HCC patients treated with curative surgical resection, RFA, TACE, or systemic chemotherapy. In addition to the pre-treatment serum CRP/ALB or ALB/CRP ratio, a high post-treatment serum, high-sensitivity CRP (hsCRP)/ALB ratio was shown in a study conducted by Oh et al. [48] to independently predict worse OS and RFS in HCC patients receiving curative surgical resection.

Overall, these studies indicate that a high pre-treatment or post-treatment serum CRP/ALB ratio is a valuable independent biomarker for prediction of poor outcomes in HCC patients after treatment with various therapies.

## 5. Prognostic Significance of Serum ALB/Globulin (GLB) Ratio in HCC Patients

The ratio of the pre-treatment serum ALB level to serum GLB level is another serum ALB-based biomarker with prognostic value in HCC patients that has been validated in multiple lines of studies (Table 4). GLB is another abundant blood protein that consists of hundreds of serum globular proteins including enzymes, complement, carrier proteins, and immunoglobulins [49]. A study conducted by Deng et al. [50] showed that a low pre-treatment serum ALB/GLB ratio independently predicted worse OS and greater recurrence in HCC patients undergoing curative surgical resection. Studies conducted by Zhang et al. [51] and Utsumi et al. [52] revealed that a low pre-treatment serum ALB/GLB ratio was independently correlated with worse OS and RFS in HCC patients undergoing curative surgical resection. Another study conducted by Zhang et al. [53] verified an independent association between a low pre-treatment serum ALB/GLB ratio and worse OS in HCC patients receiving curative surgical resection or liver transplantation. Consistent with the aforementioned findings, a study conducted by Shimizu et al. [54] demonstrated that a high pre-treatment serum GLB/ALB ratio was an independent factor which predicted worse OS in HCC patients receiving curative surgical resection.

Altogether, these studies propose that a low pre-treatment serum ALB/GLB ratio holds a valuable independent prognostic value for poor outcomes in HCC patients after treatment with various therapies.

## 6. Prognostic Significance of Serum ALB–BILirubin (BIL) Grade in HCC Patients

The prognostic value of the pre-treatment serum ALB–BIL grade, which is another serum ALB-based biomarker, in HCC patients has been validated in multiple lines of studies (Table 5). BIL is a major end-product of heme catabolism in the systemic circulation and is an essential component of bile [55]. Studies conducted by Ma et al. [56], Wang et al. [57], Li et al. [58], Zhang et al. [59], Ho et al. [60], Ruzzenente et al. [61], Zhao et al. [62], Chen et al. [63], Mao et al. [64], and Tsai et al. [65] showed that a high pre-treatment serum ALB–BIL grade independently predicted worse OS and RFS in HCC patients undergoing curative surgical resection. Studies conducted by Lee et al. [66] and Fagenson et al. [67] revealed that a high pre-treatment serum ALB–BIL grade was independently correlated with greater early recurrence (≤1 year) and 30-day mortality in HCC patients undergoing curative surgical resection. Additionally, studies conducted by Bernardi et al. [68] and Liao et al. [69] identified a high pre-treatment serum ALB–BIL grade as an independent predictor for worse OS and RFS in HCC patients undergoing liver transplantation. Studies conducted by Lo et al. [70] and Murray et al. [71] ascertained an independent association between a high pre-treatment serum ALB–BIL grade and worse OS in HCC patients treated with stereotactic body radiation therapy (SBRT) and stereotactic ablative radiation therapy (SABR). Studies conducted by Kao et al. [72], An et al. [73], Chen et al. [74], and Long et al. [75] showed that a high pre-treatment serum ALB–BIL grade was a factor that was independently associated with worse OS and RFS in HCC patients treated with RFA or PMWA. Studies conducted by Hickey et al. [76], Gui et al. [77], Kim et al. [78], Antkowiak et al. [79], Khalid et al. [80], Lee et al. [81], Zhong et al. [82], Ho et al. [83], and Chen et al. [84] confirmed that a high pre-treatment serum ALB–BIL grade was an independent factor which predicted worse OS in HCC patients receiving TACE or transarterial radioembolization (TARE). Furthermore, studies conducted by Kuo et al. [85], Nguyen et al. [86], and Tada et al. [87] showed that a high pre-treatment serum ALB–BIL grade independently predicted worse OS in HCC patients receiving molecular targeted therapy with sorafenib. Studies conducted by Ho et al. [88], Ho et al. [89], Ho et al. [90], Chang et al. [91], Ho et al. [92], and Ko et al. [93] demonstrated that a high pre-treatment serum ALB–BIL grade was independently correlated with worse OS in HCC patients treated with curative surgical resection, liver transplantation, radiotherapy, RFA, PEI, TACE, molecular targeted therapy, or supportive care. Another study conducted by Kim et al. [94] revealed that a high pre-treatment serum ALB–BIL grade was a factor that was independently associated with worse OS and progression-free survival (PFS) in HCC patients treated with proton beam therapy (PBT).

Moreover, a study conducted by Huang et al. [95] showed that a high pre-treatment serum ALB–BIL grade independently predicted worse OS in HCC patients treated with TACE combined with cryoablation (CRA). Studies conducted by Wang et al. [96] and Zhong et al. [97] found that a high pre-treatment serum ALB–BIL grade was independently associated with worse OS in HCC patients receiving TACE combined with molecular targeted therapy with sorafenib. A study conducted by Dong et al. [98] demonstrated that a high pre-treatment serum ALB–BIL grade was an independent predictor for worse OS and RFS in HCC patients receiving hypofractionated radiation therapy (HFRT) or SBRT combined with immunotherapy with camrelizumab or sintilimab.

In addition, a study conducted by Ho et al. [99] showed that a high pre-treatment serum easy ALB–BIL grade independently predicted worse OS in HCC patients treated with curative surgical resection, liver transplantation, local ablation therapy, TACE, systemic chemotherapy, molecular targeted therapy, or supportive care. Another study conducted by Tada et al. [100] revealed that a high pre-treatment serum modified ALB–BIL grade was a factor that was independently correlated with worse OS in HCC patients receiving molecular targeted therapy with lenvatinib.

Other than the pre-treatment serum ALB–BIL grade, a high on-treatment serum ALB–BIL grade was shown in a study conducted by Wang et al. [101] to independently predict worse OS in HCC patients receiving molecular targeted therapy with sorafenib followed by regorafenib. Furthermore, studies conducted by Amisaki et al. [102], Cho et al. [103], and Lin et al. [104] confirmed that a high post-treatment serum ALB–BIL grade was an independent factor which predicted worse OS and RFS in HCC patients undergoing curative surgical resection. Another study conducted by Lee et al. [105] verified an independent association between a high post-treatment serum ALB–BIL grade and worse post-progression survival (PPS) in HCC patients who received molecular targeted therapy with sorafenib and developed progressive disease (PD).

Moreover, studies conducted by Li et al. [106] and Ye et al. [107] revealed that an increased or high post-treatment serum ALB–BIL grade change was independently correlated with worse OS and RFS in HCC patients undergoing curative surgical resection. A study conducted by Lin et al. [108] identified increased post-treatment serum ALB–BIL grade change as an independent predictor for worse OS and greater recurrence in HCC patients treated with TACE. Another study conducted by Unome et al. [109] showed that a high post-treatment serum ALB–BIL grade change was a factor that was independently associated with worse OS in HCC patients receiving molecular targeted therapy with atezolizumab and bevacizumab.

Collectively, these studies suggest that a high pre-treatment, on-treatment, or post-treatment serum ALB–BIL grade is a significant independent prognostic biomarker for poor outcomes in HCC patients after treatment with various therapies.

## 7. Prognostic Significance of Serum Platelet (PLT)-ALB–BIL Grade in HCC Patients

On the basis of serum ALB–BIL grade, the pre-treatment serum PLT–ALB–BIL grade has been developed as another serum ALB-based biomarker and has been validated with prognostic value in HCC patients in multiple lines of studies (Table 6). PLT plays a critical role in not only hemostasis, thrombosis, and wound healing, but also inflammatory response and immune regulation [110]. Studies conducted by Luo et al. [111], Lu et al. [112], Wu et al. [113], and Pang et al. [114] showed that a high pre-treatment serum PLT–ALB–BIL grade independently predicted worse OS and RFS in HCC patients undergoing curative surgical resection. A study conducted by Ho et al. [115] revealed that a high pre-treatment serum PLT–ALB–BIL grade was independently correlated with worse OS in HCC patients treated with hypofractionated radiation therapy (HFRT). A study conducted by Carling et al. [116] ascertained that a high pre-treatment serum PLT–ALB–BIL grade was independently associated with worse OS in HCC patients treated with drug-eluting embolic TACE (DEE-TACE). Another study conducted by Lee et al. [117] demonstrated an independent association between a high pre-treatment serum PLT–ALB–BIL grade and worse OS in HCC patients receiving curative surgical resection, RFA, TACE, or supportive care. Furthermore, studies conducted by Ni et al. [118] and Hu et al. [119] identified a high pre-treatment serum PLT–ALB–BIL grade as an independent predictor for worse OS in HCC patients receiving TACE combined with PMWA or molecular targeted therapy with sorafenib. In addition to the pre-treatment serum PLT–ALB–BIL grade, a non-increased post-treatment serum PLT–ALB–BIL grade change was shown in a study conducted by Wang et al. [120] to be an independent factor which predicted worse OS and RFS in HCC patients treated with curative surgical resection.

Taken together, these studies suggest that a high pre-treatment or post-treatment serum PLT–ALB–BIL grade is a significant independent biomarker for prediction of poor outcomes in HCC patients after treatment with various therapies.

## 8. Prognostic Significance of Other Serum ALB-Based Mono-Biomarkers in HCC Patients

The prognostic value of various other pre-treatment serum ALB-based mono-biomarkers in HCC patients has been validated in multiple lines of studies (Table 7). Studies conducted by Xu et al. [121] and Mai et al. [122] showed that a high pre-treatment serum fibrinogen (FIB)/ALB ratio was an independent predictor for worse OS and DFS in HCC patients undergoing curative surgical resection. Studies conducted by Shen et al. [123] and Liu et al. [124] revealed that a low pre-treatment serum ALB/gamma-glutamyltransferase (GGT) ratio was independently correlated with worse OS and RFS in HCC patients receiving curative surgical resection or RFA. Consistent with the findings of the aforementioned studies, studies conducted by Liu et al. [125] and Zhang et al. [126] verified an independent association between a high pre-treatment serum GGT/ALB ratio and worse OS and DFS in HCC patients treated with curative surgical resection. Furthermore, studies conducted by Gan et al. [127], Haruki et al. [128], Li et al. [129], Meira Junior et al. [130], Shen et al. [131], and Iida et al. [132] demonstrated that a high pre-treatment serum lactic dehydrogenase (LDH)/ALB ratio, serum prothrombin time–international normalized ratio (PT-INR)/ALB ratio, serum PLT/ALB ratio, serum PLT–ALB grade, serum neutrophil to lymphocyte ratio (NLR)/ALB ratio, and a low pre-treatment serum CRP-ALB-lymphocyte (LYM) index independently predicted worse OS, DFS, and RFS in HCC patients treated with curative surgical resection. Another study conducted by Peng et al. [133] revealed that a high pre-treatment serum aspartate aminotransferase (AST)/ALB ratio was a factor that was independently correlated with worse OS and RFS in HCC patients undergoing curative surgical resection.

Overall, these studies support that serum ALB-based mono-biomarkers have a valuable and independent prognostic value for poor outcomes in HCC patients after treatment with various therapies.

## 9. Prognostic Significance of Serum ALB-Based Combination Biomarkers in HCC Patients

Multiple lines of studies have validated the prognostic value of combinations of various pre-treatment serum ALB-based biomarkers and other biomarkers in HCC patients (Table 8). A study conducted by Li et al. [134] showed that a high score of pre-treatment serum ALB–BIL grade combined with a pre-treatment serum PLT/LYM ratio independently predicted worse OS and RFS in HCC patients undergoing curative surgical resection. Studies conducted by Liao et al. [135] and Zhang et al. [136] revealed that a high score or grade of a pre-treatment serum ALB–BIL combined with pre-treatment fibrosis-4 (FIB-4) score was independently correlated with worse OS and RFS in HCC patients undergoing curative surgical resection. Another study conducted by Luo et al. [137] verified that a high score of pre-treatment serum ALB–BIL grade combined with pre-treatment serum AST/PLT ratio was independently associated with worse OS and RFS in HCC patients receiving curative surgical resection. Additionally, a study conducted by Zhang et al. [138] revealed that a high pre-treatment serum ALB–BIL grade combined with pre-treatment serum GGT level was an independent factor which predicted worse OS and DFS in HCC patients receiving curative surgical resection. Studies conducted by Pan et al. [139] and Liang et al. [140] showed that a high score or grade of the pre-treatment serum ALB–BIL combined with pre-treatment prognostic nutritional index (PNI) independently predicted worse OS and DFS in HCC patients treated with curative surgical resection or RFA. Studies conducted by Yang et al. [141] and Ha et al. [142] ascertained that a high grade or score of the pre-treatment serum ALB–BIL combined withpre-treatment systemic immune-inflammation index (SII) was independently correlated with worse OS, RFS, and cancer-specific survival (CSS) in HCC patients treated with RFA, PMWA, or TACE. A study conducted by Qin et al. [143] identified a high pre-treatment serum ALB–BIL grade combined with pre-treatment clinically significant portal hypertension (CSPH) as an independent predictor for worse OS and RFS in HCC patients undergoing curative surgical resection.

Moreover, a study conducted by Kaibori et al. [144] revealed that a high score of the pre-treatment serum modified ALB–BIL grade combined with pre-treatment serum AFP level was a factor that was independently correlated with worse OS and RFS in HCC patients treated with curative surgical resection. A study conducted by Hatanaka et al. [145] ascertained that a high score of the pre-treatment serum modified ALB–BIL grade combined with pre-treatment serum AFP level independently predicted worse OS and PFS in HCC patients receiving molecular targeted therapy with atezolizumab and bevacizumab. Another study conducted by Campani et al. [146] showed that a high score of the pre-treatment serum ALB–BIL grade combined with post-treatment serum AFP level change was independently correlated with worse OS and PFS in HCC patients receiving molecular targeted therapy with atezolizumab and bevacizumab.

Furthermore, a study conducted by Shen et al. [147] confirmed an independent association between a high grade of the pre-treatment serum CRP/ALB ratio combined with pre-treatment serum NLR and worse OS in HCC patients treated with TACE followed by RFA. A study conducted by Zhang et al. [148] demonstrated that a high grade of the pre-treatment serum ALB/FIB ratio combined with pre-treatment serum GGT/PLT ratio was independently associated with worse OS and RFS in HCC patients receiving curative surgical resection. Another study conducted by Jeng et al. [149] verified that a high grade of the pre-treatment serum ALB level combined with a pre-treatment hepatitis B virus (HBV) pre-S2 gene deletion mutation as an independent predictor for worse RFS in HCC patients undergoing curative surgical resection.

Altogether, these studies indicate that serum ALB-based combination biomarkers hold a significant and independent prognostic value for poor outcomes in HCC patients after treatment with various therapies.

## 10. Conclusions

This review provides a comprehensive summary of the up-to-date published literature that validates the independent prognostic significance of pre-treatment, on-treatment, or post-treatment serum ALB level and various ALB-based mono- and combination biomarkers in predicting the prognosis of HCC patients undergoing different surgical, locoregional, and systemic treatments. Considering that different HCC patient groups may have distinct clinicopathological backgrounds and treatment modalities, it is quite important to choose the type of ALB-based biomarker which exhibits the most optimal prognostic performance for individual HCC patients in order to achieve the best prediction of patient outcomes after therapy. Moreover, since ALB has been commonly applied in clinical settings as a powerful biomaterial and therapeutic agent for the treatment of a broad range of human diseases [16,17,18,19], identification of HCC patients with poor prognosis by ALB-based biomarkers may hold great promise in selecting the patients suitable for receiving ALB-based therapeutic strategies.

In addition, the molecular structure of ALB has been shown to undergo extensive damage under conditions of chronic-liver-disease-related liver failure such as decompensated cirrhosis due to systemic inflammation and oxidative stress, leading to a decline in the functional capacity of ALB [150,151]. Furthermore, many post-translational modifications of ALB, including phosphorylation, glycation, methylation, carbonylation, and acetylation, have been identified and display different potential impacts on ALB functions [152,153]. These structural and post-translational alterations of ALB may possibly explain the complexity of the use of serum ALB level in medical applications and may hold a great promise to discover novel prognostic value of serum ALB in HCC patients.

## Figures and Tables

**Table 1:** Prognostic significance of serum ALB level in HCC patients.

Biomarkers	Patients and Treatment Modalities	Prognostic Significance	Year	References
Pre-treatment ALB level	145 patients with small HCC (≤3 cm) treated with curative surgical resection.	Low ALB level predicted worse OS (<3.7 vs. ≥3.7 g/dL).	2003	Chen et al. [20]
	234 patients with noncirrhotic HCC treated with curative surgical resection.	Low ALB level predicted worse OS and DFS (≤3.5 vs. >3.5 g/dL).	2003	Chen et al. [21]
	303 patients with HCC treated with curative surgical resection.	Low ALB level predicted worse OS and RFS (≤4 vs. >4 g/dL).	2019	Wang et al. [22]
	136 patients with HCC treated with EBRT.	Low ALB level predicted worse OS (≤3.5 vs. >3.5 g/dL).	2008	Zeng et al. [23]
	71 cirrhotic patients with HCC treated with PEI.	Low ALB level predicted worse OS (≤3 vs. >3 g/dL).	1997	Castellano et al. [24]
	100 patients with HCC treated with PEI.	Low ALB level predicted worse OS (≤3.5 vs. >3.5 g/dL).	2002	Kuriyama et al. [25]
	137 patients with HCC treated with RFA or PMWA.	Low ALB level predicted worse OS (≤3.5 vs. >3.5 g/dL).	2005	Xu et al. [26]
	100 HCV-infected patients with small HCC (≤3 cm) treated with RFA.	Low ALB level predicted worse OS (<3.5 vs. ≥3.5 g/dL).	2012	Toshikuni et al. [27]
	128 patients with HCC treated with TAE.	Low ALB level predicted worse OS (≤3.5 vs. >3.5 g/dL).	2002	Ikeda et al. [28]
	320 patients with HCC treated with TACE.	Low ALB level predicted worse OS (≤3.5 vs. >3.5 g/dL).	2003	O’Suilleabhain et al. [29]
	141 patients with HCC treated with curative surgical resection, PEI, TACE, or antihormonal therapy.	Low ALB level predicted worse OS (≤3.3 vs. >3.3 g/dL).	2001	Lerose et al. [30]
	201 patients with HCC treated with molecular targeted therapy with sorafenib.	Low ALB level predicted worse OS and FFS (<3.4 vs. ≥3.4 g/dL).	2011	Baek et al. [31]

Abbreviations: ALB—albumin; HCC—hepatocellular carcinoma; PEI—percutaneous ethanol injection; OS—overall survival; TACE—transarterial chemoembolization; TAE—transarterial embolization; DFS—disease-free survival; RFA—radiofrequency ablation; PMWA—percutaneous microwave ablation; EBRT—external beam radiation therapy; FFS—failure-free survival; HCV—hepatitis C virus; RFS—recurrence-free survival; vs.—versus.

**Table 2:** Prognostic significance of serum ALB/ALP ratio in HCC patients.

Biomarkers	Patients	Prognostic Significance	Year	References
Pre-treatment ALB/ALP ratio ^a^	217 patients with HCC treated with curative surgical resection (as training cohort); 256 patients with HCC treated with curative surgical resection; and 425 patients with HCC treated with transarterial therapy, systemic chemotherapy, or supportive care (as 2 validation cohorts).	Low ALB/ALP ratio predicted worse OS and DFS (<0.23 vs. ≥0.23).	2015	Chan et al. [33]
	221 HBV-infected patients with HCC treated with curative surgical resection.	Low ALB/ALP ratio predicted worse OS and RFS (≤0.40 vs. >0.40).	2020	Li et al. [34]
	267 patients with combined HCC and cholangiocarcinoma treated with curative surgical resection (187 as training cohort and 80 as validation cohort).	Low ALB/ALP ratio predicted worse OS (≤0.43 vs. >0.43).	2020	Zhang et al. [35]
	210 patients with HCC treated with liver transplantation.	Low ALB/ALP ratio predicted worse OS (≤0.38 vs. >0.38).	2020	Li et al. [36]
	445 patients with HCC treated with RFA (297 as training cohort and 148 as validation cohort).	Low ALB/ALP ratio predicted worse OS and RFS (≤0.40 vs. >0.40).	2021	Zhang et al. [37]
	372 patients with HCC treated with TACE (as training cohort); 82 patients with HCC treated with TACE; and 202 patients with HCC treated with supportive care (as 2 validation cohorts).	Low ALB/ALP ratio predicted worse OS (≤0.439 vs. >0.439).	2018	Chen et al. [38]
	237 patients with advanced HCC treated without any standard anti-cancer therapies.	Low ALB/ALP ratio predicted worse OS (≤0.38 vs. >0.38).	2018	Cai et al. [39]

^a^ ALB/ALP ratio was calculated by dividing serum ALB level (g/L) by serum ALP level (IU/L). Abbreviations: ALB—albumin; ALP—alkaline phosphatase; HCC—hepatocellular carcinoma; OS—overall survival; DFS—disease-free survival; TACE—transarterial chemoembolization; HBV—hepatitis B virus; RFS—recurrence-free survival; RFA—radiofrequency ablation; vs.—versus.

**Table 3:** Prognostic significance of serum CRP/ALB ratio in HCC patients.

Biomarkers	Patients	Prognostic Significance	Year	References
Pre-treatment CRP/ALB ratio ^a^	239 patients with HCC treated with curative surgical resection.	High CRP/ALB ratio predicted worse OS (>0.028 vs. ≤0.028).	2018	Shimizu et al. [41]
	187 patients with HCC treated with curative surgical resection.	High CRP/ALB ratio predicted worse TFS (≥0.037 vs. <0.037).	2019	Ren et al. [42]
	186 patients with HCC treated with curative surgical resection, RFA, PEI, or TACE.	High CRP/ALB ratio predicted worse OS (≥0.037 vs. <0.037).	2015	Kinoshita et al. [43]
	979 patients with HCC treated with curative surgical resection, RFA, or TACE (659 as training cohort and 320 as validation cohort).	High CRP/ALB ratio predicted worse OS (≥0.05 vs. <0.05).	2018	Chen et al. [44]
	958 patients with HCC treated with TACE.	High CRP/ALB ratio predicted worse OS (≥0.06 vs. <0.06).	2022	Li et al. [45]
	522 patients with HCC treated with molecular targeted therapy with lenvatinib.	High CRP/ALB ratio predicted worse OS (≥0.108 vs. <0.108).	2022	Tada et al. [46]
Pre-treatment ALB/CRP ratio ^b^	409 patients with HCC treated with curative surgical resection, RFA, TACE, or systemic chemotherapy.	Low ALB/CRP ratio predicted worse OS and DFS (≤5.4 vs. >5.4).	2019	Wu et al. [47]
Post-treatment hsCRP/ALB ratio ^c^	389 patients with HCC treated with curative surgical resection.	High hsCRP/ALB ratios predicted worse OS and RFS (>0.625 and >0.500 vs. ≤0.625 and ≤0.500, respectively).	2018	Oh et al. [48]

^a^ CRP/ALB ratio was calculated by dividing serum CRP level (mg/L) by serum ALB level (g/L). ^b^ ALB/CRP ratio was calculated by dividing serum ALB level (g/L) by serum CRP level (mg/L). ^c^ Post-treatment hsCRP/ALB ratio was calculated by dividing hsCRP level (mg/L) by ALB level (g/L) using data on day 0 or day 1 after treatment. Abbreviations: CRP—C-reactive protein; ALB—albumin; HCC—hepatocellular carcinoma; RFA—radiofrequency ablation; PEI—percutaneous ethanol injection; TACE—transarterial chemoembolization; OS—overall survival; hsCRP—high-sensitivity C-reactive protein; RFS—recurrence-free survival; TFS—tumor-free survival; DFS—disease-free survival; vs.—versus.

**Table 4:** Prognostic significance of serum ALB/GLB ratio in HCC patients.

Biomarkers	Patients	Prognostic Significance	Year	References
Pre-treatment ALB/GLB ratio ^a^	172 patients with HCC treated with curative surgical resection.	Low ALB/GLB ratio predicted worse OS and greater recurrence (<1.48 vs. ≥1.48).	2016	Deng et al. [50]
	693 patients with HCC treated with curative surgical resection.	Low ALB/GLB ratio predicted worse OS and RFS (<1.0 vs. ≥1.0).	2020	Zhang et al. [51]
	157 patients with HCC treated with curative surgical resection.	Low ALB/GLB ratio predicted worse OS (<1.16 vs. ≥1.16).	2021	Utsumi et al. [52]
	150 patients with HCC treated with curative surgical resection or liver transplantation.	Low ALB/GLB ratio predicted worse OS (<1.18 vs. ≥1.18).	2016	Zhang et al. [53]
Pre-treatment GLB/ALB ratio ^b^	368 patients with HCC treated with curative surgical resection.	High GLB/ALB ratio predicted worse OS (>0.918 vs. ≤0.918)	2017	Shimizu et al. [54]

^a^ ALB/GLB ratio was calculated by dividing serum ALB level (g/L) by serum GLB level (g/L). ^b^ GLB/ALB ratio was calculated by dividing serum GLB level (g/L) by serum ALB level (g/L). Abbreviations: ALB—albumin; GLB—globulin; HCC—hepatocellular carcinoma; OS—overall survival; RFS—recurrence-free survival; vs.—versus.

**Table 5:** Prognostic significance of serum ALB–BIL grade in HCC patients.

Biomarkers	Patients	Prognostic Significance	Year	References
Pre-treatment ALB–BIL grade ^a^	318 patients with HCC treated with curative surgical resection (160 as training cohort and 158 as validation cohort).	High ALB–BIL grade predicted worse OS (grade 2 vs. 1).	2016	Ma et al. [56]
	1242 patients with HCC treated with curative surgical resection.	High ALB–BIL grade predicted worse OS (grade 2 vs. 1).	2016	Wang et al. [57]
	491 patients with HCC treated with curative surgical resection.	High ALB–BIL grade predicted worse OS (grade 2 vs. 1).	2017	Li et al. [58]
	338 patients with HCC treated with curative surgical resection.	High ALB–BIL grade predicted worse OS (grade 2 vs. 1).	2018	Zhang et al. [59]
	1038 patients with HCC treated with curative surgical resection.	High ALB–BIL grade predicted worse RFS (grade 2/3 vs. 1).	2019	Ho et al. [60]
	187 patients with HCC treated with curative surgical resection.	High ALB–BIL grade predicted worse OS (grade 2 vs. 1).	2019	Ruzzenente et al. [61]
	196 patients with HCC treated with curative surgical resection.	High ALB–BIL grade predicted worse OS (grade 2 vs. 1).	2020	Zhao et al. [62]
	265 patients with HCC treated with curative surgical resection.	High ALB–BIL grade predicted worse OS (grade 2 vs. 1).	2021	Chen et al. [63]
	166 AFP-negative patients with HCC treated with curative surgical resection.	High ALB–BIL grade predicted worse RFS (grade 2 vs. 1).	2021	Mao et al. [64]
	2137 patients with HCC treated with curative surgical resection.	High ALB–BIL grade predicted worse OS (grade 2/3 vs. 1).	2021	Tsai et al. [65]
	465 patients with HCC treated with curative surgical resection.	High ALB–BIL grade (grade 2 or 3) independently predicted greater early recurrence (≤1 year) than low grade (grade 2/3 vs. 1).	2018	Lee et al. [66]
	13,783 patients with HCC treated with curative surgical resection.	High ALB–BIL grade predicted greater 30-day mortality (grade 2/3 vs. 1).	2020	Fagenson et al. [67]
	301 patients with HCC treated with liver transplantation.	High ALB–BIL grade predicted worse OS (grade 3 vs. 1).	2019	Bernardi et al. [68]
	75 patients with HCC treated with liver transplantation.	High ALB–BIL grade predicted worse OS and RFS (grade 2/3 vs. 1).	2019	Liao et al. [69]
	152 patients with HCC treated with SABR.	High ALB–BIL grade predicted worse OS (grade 2 vs. 1).	2017	Lo et al. [70]
	152 patients with HCC treated with SBRT.	High ALB–BIL grade predicted worse OS (grade 2 vs. 1).	2018	Murray et al. [71]
	622 patients with HCC treated with RFA.	High ALB–BIL grade predicted worse OS (grade 2/3 vs. 1).	2017	Kao et al. [72]
	183 HCV-infected patients with HCC treated with PMWA.	High ALB–BIL grade predicted worse OS (grade 2/3 vs. 1).	2019	An et al. [73]
	271 patients with HCC treated with RFA.	High ALB–BIL grade predicted worse OS and RFS (grade 2/3 vs. 1).	2019	Chen et al. [74]
	344 patients with HCC treated with RFA.	High ALB–BIL grade predicted worse OS and RFS (grade 2 vs. 1).	2022	Long et al. [75]
	765 patients with HCC treated with TACE or TARE.	High ALB–BIL grade predicted worse OS (grade 3 vs. 2).	2016	Hickey et al. [76]
	117 patients with HCC treated with TARE.	High ALB–BIL grade predicted worse OS (grade 2 vs. 1).	2018	Gui et al. [77]
	476 patients with HCC treated with TACE.	High ALB–BIL grade predicted worse OS (grade 2/3 vs. 1).	2018	Kim et al. [78]
	1000 patients with HCC treated with TARE.	High ALB–BIL grade predicted worse OS (grade 2/3 vs. 1).	2019	Antkowiak et al. [79]
	71 patients with HCC treated with TACE.	High ALB–BIL grade predicted worse OS (grade 2/3 vs. 1).	2019	Khalid et al. [80]
	293 patients with HCC treated with TACE.	High ALB–BIL grade predicted worse OS (grade 2/3 vs. 1).	2019	Lee et al. [81]
	838 patients with HCC treated with TACE (548, 115, and 175 as training cohort and 2 validation cohorts, respectively).	High ALB–BIL grade predicted worse OS (grade 3 vs. 1).	2019	Zhong et al. [82]
	1051 patients with HCC treated with TACE.	High ALB–BIL grade predicted worse OS (grade 2 vs. 1).	2021	Ho et al. [83]
	359 patients with HCC treated with TACE.	High ALB–BIL grade predicted worse OS (grade 2/3 vs. 1).	2022	Chen et al. [84]
	260 patients with HCC treated with molecular targeted therapy with sorafenib.	High ALB–BIL grade predicted worse OS (grade 2 vs. 1).	2017	Kuo et al. [85]
	110 patients with HCC treated with molecular targeted therapy with sorafenib.	High ALB–BIL grade predicted worse OS (grade 3 vs. 1/2).	2019	Nguyen et al. [86]
	567 patients with HCC treated with molecular targeted therapy with sorafenib.	High ALB–BIL grade predicted worse OS (grade 2/3 vs. 1).	2019	Tada et al. [87]
	829 patients with HCC treated with curative surgical resection, local ablation therapy, or TACE.	High ALB–BIL grade predicted worse OS (grade 2/3 vs. 1).	2019	Ho et al. [88]
	1846 patients with HCC treated with curative surgical resection, liver transplantation, radiotherapy, local ablation therapy, TACE, molecular targeted therapy, or supportive care.	High ALB–BIL grade predicted worse OS (grade 2/3 vs. 1).	2020	Ho et al. [89]
	1093 patients with HCC treated with curative surgical resection, liver transplantation, local ablation therapy, TACE, systemic chemotherapy, molecular targeted therapy, or supportive care.	High ALB–BIL grade predicted worse OS (grade 2/3 vs. 1).	2020	Ho et al. [90]
	420 patients with HCC treated with curative surgical resection, RFA, PEI, TACE, or supportive care.	High ALB–BIL grade predicted worse OS (grade 2/3 vs. 1).	2021	Chang et al. [91]
	1898 patients with HCC treated with curative surgical resection, liver transplantation, local ablation therapy, TACE, molecular targeted therapy, or supportive care.	High ALB–BIL grade predicted worse OS (grade 2/3 vs. 1).	2022	Ho et al. [92]
	3341 HBV- and/or HCV-infected patients with HCC treated with curative surgical resection, liver transplantation, local ablation therapy, or TACE.	High ALB–BIL grade predicted worse OS (grade 2/3 vs. 1).	2022	Ko et al. [93]
	46 patients with HCC treated with PBT.	High ALB–BIL grade predicted worse OS and PFS (grade 2/3 vs. 1).	2022	Kim et al. [94]
	86 patients with HCC (>4 cm) treated with TACE combined with CRA.	High ALB–BIL grade predicted worse OS (grade 2/3 vs. 1).	2020	Huang et al. [95]
	173 patients with HCC treated with TACE combined with molecular targeted therapy with sorafenib.	High ALB–BIL grade predicted worse OS (grade 2 vs. 1).	2020	Wang et al. [96]
	504 patients with HCC treated with TACE combined with molecular targeted therapy with sorafenib (319, 61, and 124 as training cohort and 2 validation cohorts, respectively).	High ALB–BIL grade predicted worse OS (grade 2 vs. 1).	2020	Zhong et al. [97]
	38 patients with HCC treated with HFRT or SBRT combined with immunotherapy with camrelizumab or sintilimab.	High ALB–BIL grade predicted worse OS and RFS (grade 2 vs. 1).	2022	Dong et al. [98]
Pre-treatment easy ALB–BIL grade ^b^	3794 patients with HCC treated with curative surgical resection, liver transplantation, local ablation therapy, TACE, systemic chemotherapy, molecular targeted therapy, or supportive care.	High easy ALB–BIL grade predicted worse OS (grade 2/3 vs. 1).	2021	Ho et al. [99]
Pre-treatment modified ALB–BIL grade ^c^	524 patients with HCC treated with molecular targeted therapy with lenvatinib.	High modified ALB–BIL grade predicted worse OS (grade 2b/3 vs. 1/2a).	2021	Tada et al. [100]
On-treatment ALB–BIL grade ^d^	88 patients with HCC treated with molecular targeted therapy with sorafenib followed by regorafenib.	High ALB–BIL grade predicted worse OS (grade 2 vs. 1).	2021	Wang et al. [101]
Post-treatment ALB–BIL grade	136 patients with HCC treated with curative surgical resection.	High ALB–BIL grade predicted worse OS and RFS (grade 3 vs. 2) ^e^.	2018	Amisaki et al. [102]
	525 patients with HCC treated with curative surgical resection.	High ALB–BIL grade predicted worse OS and RFS (grade 2/3 vs. grade 1) ^f^.	2020	Cho et al. [103]
	383 patients with HCC treated with curative surgical resection.	High ALB–BIL grade predicted worse OS and RFS (grade 2/3 vs. 1) ^g^.	2020	Lin et al. [104]
	310 patients with HCC treated with molecular targeted therapy with sorafenib and developing PD (155 as training cohort and 155 as validation cohort).	High ALB–BIL grade predicted worse PPS (grade 3 vs. 1).	2018	Lee et al. [105]
Post-treatment ALB–BIL grade change	258 HBV-infected patients with HCC treated with curative surgical resection	Increased ALB–BIL grade change predicted worse OS and greater recurrence (>0 vs. ≤0) ^h^.	2018	Li et al. [106]
	300 patients with HCC treated with curative surgical resection.	High ALB–BIL grade change predicted worse OS and RFS (>0.71 vs. ≤0.71) ^i^.	2019	Ye et al. [107]
	613 HCV-infected patients with HCC treated with TACE.	Increased ALB–BIL grade change predicted worse OS and greater recurrence (>0 vs. ≤0) ^j^.	2022	Lin et al. [108]
	69 patients with HCC treated with molecular targeted therapy with atezolizumab and bevacizumab.	High ALB–BIL grade change predicted worse OS (>0.376 vs. ≤0.376) ^k^.	2022	Unome et al. [109]

^a^ ALB–BIL grade was calculated by the formula 0.66 × log_10_ serum BIL level (μmol/L) − 0.085 × serum ALB level (g/L) and stratified as grade 1 (≤−2.60), grade 2 (>−2.60 to ≤−1.39), or grade 3 (>−1.39). ^b^ Easy ALB–BIL grade was calculated as the formula serum BIL (mg/dL) − 9 × serum ALB level (g/dL) and stratified as grade 1 (≤−34.4), grade 2 (>−34.4 to <−22.2), or grade 3 (≥−22.2). ^c^ Modified ALB–BIL grade was calculated by the same formula as ALB–BIL grade and stratified as grade 1 (≤ –2.60), grade 2a (>−2.60 to ≤−2.27), grade 2b (>−2.27 to ≤−1.39), or grade 3 (>−1.39). ^d^ On-treatment ALB–BIL grade was calculated using data from before the initiation of regorafenib therapy. ^e^ Post-treatment ALB–BIL grade was calculated using data from day 5 after treatment. ^f^ Post-treatment ALB–BIL grade was calculated using data from the 1st year after treatment. ^g^ Post-treatment ALB–BIL grade was calculated using data from the 5th year after treatment. ^h^ Post-treatment ALB–BIL grade change was calculated by subtracting pre-treatment ALB–BIL grade from post-treatment 1st-month ALB–BIL grade. ^i^ Post-treatment ALB–BIL grade change was calculated by subtracting pre-treatment ALB–BIL grade from the post-treatment 1st-day ALB–BIL grade. ^j^ Post-treatment ALB–BIL grade change was calculated by subtracting pre-treatment ALB–BIL grade from post-first-round-TACE ALB–BIL grade. ^k^ Post-treatment ALB–BIL grade change was calculated by subtracting pre-treatment ALB–BIL grade from post-treatment 3rd-month ALB–BIL grade. Abbreviations: ALB—albumin; BIL—bilirubin; HCC—hepatocellular carcinoma; TACE—transarterial chemoembolization; TARE—transarterial radioembolization; OS—overall survival; SABR—stereotactic ablative radiation therapy; RFS—recurrence-free survival; PD—progressive disease; PPS—post-progression survival; HBV—hepatitis B virus; SBRT—stereotactic body radiation therapy; HCV—hepatitis C virus; PMWA—percutaneous microwave ablation; CRA—cryoablation; RFA—radiofrequency ablation; PEI—percutaneous ethanol injection; AFP—alpha-fetoprotein; HFRT—hypofractionated radiation therapy; PBT—proton beam therapy; PFS—progression-free survival; vs.—versus.

**Table 6:** Prognostic significance of serum PLT–ALB–BIL grade in HCC patients.

Biomarkers	Patients	Prognostic Significance	Year	References
Pre-treatment PLT–ALB–BIL grade ^a^	785 HBV-infected patients with HCC treated with curative surgical resection.	High PLT–ALB–BIL grade predicted worse OS and RFS (grade 2/3 vs. 1).	2018	Luo et al. [111]
	2038 patients with HCC treated with curative surgical resection.	High PLT–ALB–BIL grade predicted worse OS (grade 2/3 vs. 1).	2019	Lu et al. [112]
	134 HBV-infected patients with HCC treated with curative surgical resection.	High PLT–ALB–BIL grade predicted worse OS and RFS (grade 2/3 vs. 1).	2019	Wu et al. [113]
	465 HBV-infected patients with HCC treated with curative surgical resection.	High PLT–ALB–BIL grade predicted worse OS and RFS (grade 2/3 vs. 1).	2020	Pang et al. [114]
	174 patients with HCC treated with HFRT.	High PLT–ALB–BIL grade predicted worse OS (grade 3 vs. 1).	2018	Ho et al. [115]
	49 patients with HCC treated with DEE-TACE.	High PLT–ALB–BIL grade predicted worse OS (grade 2 vs. 1).	2019	Carling et al. [116]
	6507 patients with HCC treated with curative surgical resection, RFA, TACE, or supportive care.	High PLT–ALB–BIL grade predicted worse OS (grade 2/3 vs. 1).	2019	Lee et al. [117]
	349 patients with HCC treated with TACE combined with PMWA.	High PLT–ALB–BIL grade predicted worse OS (grade 2/3 vs. 1).	2019	Ni et al. [118]
	418 patients with HCC treated with TACE combined with molecular targeted therapy with sorafenib.	High PLT–ALB–BIL grade predicted worse OS (grade 3 vs. 1).	2021	Hu et al. [119]
Post-treatment PLT–ALB–BIL grade change ^b^	489 patients with HCC treated with curative surgical resection (342 as training cohort and 147 as validation cohort)	Non-increased PLT–ALB–BIL grade change predicted worse OS and RFS (≤0 vs. >0).	2021	Wang et al. [120]

^a^ PLT–ALB–BIL grade was calculated by the formula 2.02 × log_10_ serum BIL level (μmol/L) − 0.37 × [log_10_ serum BIL level (μmol/L)]^2^ − 0.04 × serum ALB level (g/L) − 3.48 × log_10_ serum PLT count (10^9^/L) + 1.01 × [log_10_ serum PLT count (10^9^/L)]^2^ and stratified as grade 1 (≤−2.53), grade 2 (>−2.53 to ≤−2.09), or grade 3 (>−2.09). ^b^ Post-treatment PLT–ALB–BIL grade change was calculated by subtracting pre-treatment PLT–ALB–BIL grade from post-treatment, 1st-month PLT–ALB–BIL grade. Abbreviations: PLT—platelet; ALB—albumin; BIL—bilirubin; HCC—hepatocellular carcinoma; HFRT—hypofractionated radiation therapy; OS—overall survival; HBV—hepatitis B virus; RFS—recurrence-free survival; DEE-TACE—drug-eluting embolic transarterial chemoembolization; RFA—radiofrequency ablation; TACE—transarterial chemoembolization; PMWA—percutaneous microwave ablation; vs.—versus.

**Table 7:** Prognostic significance of other serum ALB-based mono-biomarkers in HCC patients.

Biomarkers	Patients	Prognostic Significance	Year	References
Pre-treatment FIB/ALB ratio ^a^	151 patients with HCC treated with curative surgical resection.	High FIB/ALB ratio predicted worse OS and greater recurrence (>0.062 vs. ≤0.062).	2018	Xu et al. [121]
	1502 patients with HCC treated with curative surgical resection.	High FIB/ALB ratio predicted worse OS and DFS (>0.089 vs. ≤0.089).	2022	Mai et al. [122]
Pre-treatment ALB/GGT ratio ^b^	480 patients with HCC treated with curative surgical resection.	Low ALB/GGT ratio predicted worse OS and RFS (≤0.5 vs. >0.5).	2019	Shen et al. [123]
	394 patients with HCC treated with RFA.	Low ALB/GGT ratio predicted worse OS and RFS (≤0.63 vs. >0.63).	2021	Liu et al. [124]
Pre-treatment GGT/ALB ratio ^c^	206 patients with HCC treated with curative surgical resection.	High GGT/ALB ratio predicted worse OS (>0.946 vs. ≤0.946).	2020	Liu et al. [125]
	Cirrhotic patients with HCC treated with curative surgical resection.	High GGT/ALB ratio predicted worse OS and DFS (≥1.1733 vs. <1.1733).	2022	Zhang et al. [126]
Pre-treatment LDH/ALB ratio ^d^	1041 patients with HCC treated with curative surgical resection (768 as training cohort and 273 as validation cohort).	High LDH/ALB ratio predicted worse OS and RFS (≥5.5 vs. <5.5).	2018	Gan et al. [127]
Pre-treatment PT-INR/ALB ratio ^e^	199 patients with HCC treated with curative surgical resection.	High PT-INR/ALB ratio predicted worse OS and DFS (≥0.288 vs. <0.288).	2018	Haruki et al. [128]
Pre-treatment PLT/ALB ratio ^f^	628 patients with HCC treated with curative surgical resection.	High PLT/ALB ratio predicted worse OS and RFS (>4.8 vs. ≤4.8).	2019	Li et al. [129]
Pre-treatment PLT–ALB grade ^g^	182 patients with HCC treated with curative surgical resection.	High PLT–ALB grade predicted worse OS (grade 2/3 vs. 1).	2022	Meira Junior et al. [130]
Pre-treatment NLR/ALB ratio ^h^	169 patients with HCC treated with curative surgical resection.	High NLR/ALB ratio predicted worse OS (>0.056 vs. ≤0.056).	2021	Shen et al. [131]
Pre-treatment CRP-ALB-LYM index ^i^	651 patients with HCC treated with curative surgical resection (384 as training cohort and 267 as validation cohort).	Low CRP-ALB-LYM index predicted worse OS and RFS (<5 vs. ≥5).	2022	Iida et al. [132]
Pre-treatment AST/ALB ratio ^j^	1874 patients with HCC treated with curative surgical resection (991 as training cohort and 883 as validation cohort).	High AST/ALB ratio predicted worse OS and RFS (>1.6 vs. <0.7).	2022	Peng et al. [133]

^a^ FIB/ALB ratio was calculated by dividing serum FIB level (mg/dL) by serum ALB level (mg/dL). ^b^ ALB/GGT ratio was calculated by dividing serum ALB level (g/L) by serum GGT level (U/L). ^c^ GGT/ALB ratio was calculated by dividing serum GGT level (U/L) by serum ALB level (g/L). ^d^ LDH/ALB ratio was calculated by dividing serum PLT count (U/L) by serum ALB level (g/L). ^e^ PT-INR/ALB ratio was calculated by dividing PT-INR by serum ALB level (g/dL). ^f^ PLT/ALB ratio was calculated by dividing serum PLT count (10^9^/L) by serum ALB level (g/L). ^g^ PLT–ALB score was calculated by the formula −0.777 × serum ALB level (g/dL) −0.575 × log_10_ serum PLT count (10^10^/L) and stratified as grade 1 (≤−3.77), grade 2 (>−3.77 to ≤−3.04), or grade 3 (>−3.04). ^h^ NLR was calculated by dividing serum NEU count (10^9^/L) by serum LYM count (10^9^/L), and NLR/ALB ratio was calculated by dividing NLR by serum ALB level (g/L). ^i^ CRP-ALB-LYM index was calculated by the formula [serum ALB level (g/dL) × serum LYM count (10^9^/L)]/[serum CRP level (mg/dL) × 10^4^]. ^j^ AST/ALB ratio was calculated by dividing serum AST level (U/L) by serum ALB level (g/L). Abbreviations: ALB—albumin; HCC—hepatocellular carcinoma; FIB—fibrinogen; OS—overall survival; DFS—disease-free survival; GGT—gamma-glutamyltransferase; RFA—radiofrequency ablation; RFS—recurrence-free survival; LDH—lactic dehydrogenase; PT-INR—prothrombin time–international normalized ratio; PLT—platelet; NLR—neutrophil to lymphocyte ratio; CRP—C-reactive protein; LYM—lymphocyte; NEU—neutrophil; AST—aspartate aminotransferase; vs.—versus.

**Table 8:** Prognostic significance of serum ALB-based combination biomarkers in HCC patients.

Biomarkers	Patients	Prognostic Significance	Year	References
Pre-treatment ALB–BIL grade combined with pre-treatment PLT/LYM ratio ^a^	475 patients with HCC treated with curative surgical resection.	High score of ALB–BIL grade combined with PLT/LYM ratio (score 1 or 2) independently predicted worse OS and RFS than low score (score 0).	2018	Li et al. [134]
Pre-treatment ALB–BIL grade combined with pre-treatment FIB-4 score	350 patients with HCC treated with curative surgical resection.	High grade of ALB–BIL grade combined with FIB-4 score (grade 2, 3, or 4) independently predicted worse RFS than low grade (grade 1) ^b^	2018	Liao et al. [135]
	544 patients with HCC treated with curative surgical resection.	High score of ALB–BIL grade combined with FIB-4 score (score 2 or 3) independently predicted worse OS and RFS than low score (score 1) ^c^.	2019	Zhang et al. [136]
Pre-treatment ALB–BIL grade combined with pre-treatment AST/PLT ratio ^d^	239 patients with HCC treated with curative surgical resection.	High score of ALB–BIL grade combined with AST/PLT ratio (score 2 or 3) independently predicted worse OS and RFS than low score (score 1).	2019	Luo et al. [137]
Pre-treatment ALB–BIL grade combined with pre-treatment GGT level ^e^	520 patients with HCC treated with curative surgical resection.	High grade of ALB–BIL grade combined with GGT level (grade 2 or 3) independently predicted worse OS and DFS than low grade (grade 1).	2019	Zhang et al. [138]
Pre-treatment ALB–BIL grade combined with pre-treatment PNI	110 patients with HCC treated with RFA.	High score of ALB–BIL grade combined with PNI (score 1, 2, or 3) independently predicted worse OS than low grade (score 0) ^f^.	2020	Pan et al. [139]
	868 HBV-infected patients with HCC treated with curative surgical resection.	High grade of ALB–BIL grade combined with PNI (grade 4) independently predicted worse OS and DFS than low grade (grade 1) ^g^.	2021	Liang et al. [140]
Pre-treatment ALB–BIL grade combined with pre-treatment SII	405 patients with HCC treated with RFA or PMWA.	High grade of ALB–BIL grade combined with SII (grade 2 or 3) independently predicted worse OS, RFS, and CSS than low grade (grade 1) ^h^.	2020	Yang et al. [141]
	295 patients with HCC treated with TACE.	High score of ALB–BIL grade combined with SII (score 2) independently predicted worse OS than low score (score 0 or 1) ^i^.	2023	Ha et al. [142]
Pre-treatment ALB–BIL grade combined with pre-treatment CSPH ^j^	1679 HBV-infected patients with HCC treated with curative surgical resection.	High grade of ALB–BIL grade combined with CSPH (grade 2 or 3) independently predicted worse OS and RFS than low grade (grade 1).	2021	Qin et al. [143]
Pre-treatment modified ALB–BIL grade combined with pre-treatment AFP level ^k^	480 patients with HCC treated with curative surgical resection.	High score of modified ALB–BIL grade combined with AFP level (score 1 or 2) independently predicted worse OS and RFS than low grade (score 0).	2022	Kaibori et al. [144]
	426 patients with HCC treated with molecular targeted therapy with atezolizumab and bevacizumab (255 as training cohort and 171 as validation cohort).	High score of modified ALB–BIL grade combined with AFP level (score 1 or 2) independently predicted worse OS and PFS than low grade (score 0).	2022	Hatanaka et al. [145]
Pre-treatment ALB–BIL grade combined with post-treatment AFP level change ^l^	75 patients with HCC treated with molecular targeted therapy with atezolizumab and bevacizumab (38 as training cohort and 37 as validation cohort).	High score of ALB–BIL grade combined with AFP level change (score 2) independently predicted worse OS and PFS than low grade (score 0 or 1).	2022	Campani et al. [146]
Pre-treatment CRP/ALB ratio combined with pre-treatment NLR ^m^	172 patients with HCC treated with TACE followed by RFA.	High grade of CRP/ALB ratio combined with NLR (grade 3) independently predicted worse OS than low grade (grade 1 or 2).	2019	Shen et al. [147]
Pre-treatment ALB/FIB ratio combined with pre-treatment GGT/PLT ratio ^n^	616 patients with HCC treated with curative surgical resection.	High grade of ALB/FIB ratio combined with GGT/PLT ratio (grade 2 or 3) independently predicted worse OS and RFS than low grade (grade 1).	2020	Zhang et al. [148]
Pre-treatment ALB level combined with pre-treatment HBV pre-S2 gene mutation ^o^	75 HBV-infected patients with HCC treated with curative surgical resection.	High grade of ALB level combined with HBV pre-S2 gene mutation (grade 4) independently predicted worse RFS than low grade (grade 1).	2021	Jeng et al. [149]

^a^ ALB–BIL grade 1 (≤−2.60), 2 (>−2.60 to ≤−1.39), or 3 (>−1.39) was allocated a score of 0, 1, or 2, respectively. PLT/LYM ratio was calculated by dividing serum PLT count (10^9^/L) by serum LYM count (10^9^/L) and stratified as score 0 (<150) or score 1 (≥150). The score of ALB–BIL grade combined with PLT/LYM ratio was the summation of ALB–BIL and PLT/LYM scores. ^b^ ALB–BIL grade was categorized as low (≤−2.60) or high (>−2.60) grade. FIB-4 score was calculated by the formula [age × serum AST level (U/L)]/serum PLT count (10^9^/L) × [serum ALT level (U/L)]^1/2^ and categorized as low (≤3.25) or high (>3.25) score. The combination of ALB–BIL grade and FIB-4 score was categorized as grade 1 (low ALB–BIL grade and low FIB-4 score), grade 2 (high ALB–BIL grade but low FIB-4 score), grade 3 (low ALB–BIL grade but high FIB-4 score), or grade 4 (high ALB–BIL grade and high FIB-4 score). ^c^ ALB–BIL grade was categorized as grade 1 (≤−2.60), grade 2 (>−2.60 to ≤−1.39), or grade 3 (>−1.39). FIB-4 score was categorized as score 0 (≤3.25) or score 1 (>3.25). The score of ALB–BIL grade combined with FIB-4 score was the summation of ALB–BIL grade and FIB-4 score. ^d^ ALB–BIL grade was categorized as grade 1 (≤−2.60), grade 2 (>−2.60 to ≤−1.39), or grade 3 (>−1.39). AST/PLT ratio was calculated by the formula [serum AST level (U/L)/ULN]/serum PLT count (10^9^/L) × 100 and categorized as score 0 (<0.5) or score 1 (≥0.5). The score of ALB–BIL grade combined with AST/PLT ratio was the summation of ALB–BIL grade and AST/PLT score. ^e^ ALB–BIL grade was categorized as low (≤−2.60) or high (>−2.60) grade. GGT level was categorized as low (≤50 U/L) or high (>50 U/L) level. The combination of ALB–BIL grade and GGT level was categorized as grade 1 (low ALB–BIL grade and low GGT level), grade 2 (high ALB–BIL grade or high GGT level), or grade 3 (high ALB–BIL grade and high GGT level). ^f^ ALB–BIL grade 1 (≤−2.60), 2 (>−2.60 to ≤−1.39), or 3 (>−1.39) was allocated a score of 0, 1, or 2, respectively. PNI was calculated by the formula serum ALB level (g/L) + 5 × serum LYM count (10^9^/L) and categorized as score 0 (≥47.2) or 1 (<47.2). The score of ALB–BIL grade combined with PNI was the summation of ALB–BIL and PNI scores. ^g^ ALB–BIL grade was categorized as low (≤−2.80) or high (>−2.80) grade. PNI was categorized as low (≤46) or high (>46) score. The combination of ALB–BIL grade and PNI was categorized as grade 1 (low ALB–BIL grade but high PNI score), grade 2 (low ALB–BIL grade and low PNI score), grade 3 (high ALB–BIL grade and high PNI score), or grade 4 (high ALB–BIL grade but low PNI score). ^h^ ALB–BIL grade was categorized as low (≤−2.60) or high (>−2.60) grade. SII was calculated by the formula serum NEU count (10^9^/L) × serum PLT count (10^9^/L) / serum LYM count (10^9^/L) and categorized as low (≤330 × 10^9^) or high (>330 × 10^9^) score. The combination of ALB–BIL grade and SII was categorized as grade 1 (low ALB–BIL grade and low SII score), grade 2 (high ALB–BIL grade or high SII score), or grade 3 (high ALB–BIL grade and high SII score). ^i^ ALB–BIL grade was categorized as low (≤−2.60) or high (>−2.60) grade. SII was categorized as low (≤152.8) or high (>152.8) score. The combination of ALB–BIL grade and SII was categorized as score 0 (low ALB–BIL grade and low SII score), score 1 (high ALB–BIL grade or high SII score), or score 2 (high ALB–BIL grade and high SII score). ^j^ ALB–BIL grade was categorized as grade 1 (≤−2.60), grade 2 (>−2.60 to ≤−1.39), or grade 3 (>−1.39). CSPH was defined by the presence of esophagogastric varices and/or a serum PLT count < 100 × 10^9^/l in association with splenomegaly and categorized as score 0 or 1 for the absence or presence of CSPH, respectively. The score of ALB–BIL grade combined with CSPH was the summation of ALB–BIL grade and CSPH score. ^k^ Modified ALB–BIL grade was categorized as grade 1 (≤−2.60), grade 2a (>−2.60 to ≤−2.27), grade 2b (>−2.27 to ≤−1.39), or grade 3 (>−1.39). Serum AFP level was categorized as low (<100 ng/mL) or high (≥100 ng/mL) level. The combination of modified ALB–BIL grade and serum AFP level was categorized as score 0 (modified ALB–BIL grade 1/2a and low serum AFP ratio), score 1 (modified ALB–BIL grade 2b/3 or high serum AFP ratio), or score 2 (modified ALB–BIL grade 2b/3 and high serum AFP ratio). ^l^ ALB–BIL grade was categorized as grade 1 (≤−2.60), grade 2 (>−2.60 to ≤−1.39), or grade 3 (>−1.39). Post-treatment AFP level change was calculated by subtracting pre-treatment AFP level from post-treatment 3rd-week AFP level and categorized as decreased (≥20%) or non-decreased (<20%). The combination of ALB–BIL grade and AFP level change was categorized as score 0 (ALB–BIL grade 1 and decreased AFP level change), score 1 (ALB–BIL grade 2 or non-decreased AFP level change), or score 2 (ALB–BIL grade 2 and non-decreased AFP level change). ^m^ CRP/ALB ratio was categorized as low (≤0.275) or high (>0.275) ratio. NLR was categorized as low (≤2.205) or high (>2.205) ratio. The combination of CRP/ALB ratio and NLR was categorized as grade 1 (low CRP/ALB ratio and low NLR ratio), grade 2 (high CRP/ALB ratio or high NLR ratio), or grade 3 (high CRP/ALB ratio and high NLR ratio). ^n^ ALB/FIB ratio was calculated by dividing serum ALB level (mg/dL) by serum FIB level (mg/dL) and stratified as low (<9.6) or high (≥9.6) ratio. GGT/PLT ratio was calculated by dividing serum GGT level (U/L) by serum PLT count (10^9^/L) and categorized as low (<0.24) or high (≥0.24) ratio. The combination of ALB/FIB ratio and GGT/PLT ratio was categorized as grade 1 (high ALB/FIB ratio but low GGT/PLT ratio), grade 2 (high ALB/FIB ratio and high GGT/PLT ratio or low ALB/FIB ratio and low GGT/PLT ratio), or grade 3 (low ALB/FIB ratio but high GGT/PLT ratio). ^o^ Serum ALB level was categorized as low (≤3.8 g/dL) or high (>3.8 g/dL) level. HBV pre-S2 gene mutation was defined by the presence of deletion mutations spanning the pre-S2 gene segment of viral DNA in serum. The combination of ALB level combined with HBV pre-S2 gene mutation was categorized as grade 1 (high ALB level but absence of mutation), grade 2 (low ALB level and absence of mutation), grade 3 (high ALB level and presence of mutation), or grade 4 (low ALB level but presence of mutation). Abbreviations: ALB—albumin; HCC—hepatocellular carcinoma; BIL—bilirubin; PLT—platelet; LYM—lymphocyte; FIB-4—fibrosis-4; AST—aspartate aminotransferase; CRP—C-reactive protein; NLR—neutrophil to lymphocyte ratio; TACE—transarterial chemoembolization; RFA—radiofrequency ablation; GGT—gamma-glutamyltransferase; DFS—disease-free survival; PNI—prognostic nutritional index; SII—systemic immune-inflammation index; PMWA—percutaneous microwave ablation; CSS—cancer-specific survival; FIB—fibrinogen; NEU—neutrophil; HBV—hepatitis B virus; CSPH—clinically significant portal hypertension; ALT—alanine aminotransferase; ULN—upper limit of normal; AFP—alpha-fetoprotein; PFS—progression-free survival; vs.—versus.

## Abbreviations

HCC—hepatocellular carcinoma; ALB—albumin; OS—overall survival; DFS—disease-free survival; RFS—recurrence-free survival; EBRT—external beam radiation therapy; PEI—percutaneous ethanol injection; RFA—radiofrequency ablation; PMWA—percutaneous microwave ablation; TAE—transarterial embolization; TACE—transarterial chemoembolization; FFS—failure-free survival; ALP—alkaline phosphatase; CRP—C-reactive protein; TFS—tumor-free survival; hsCRP—high-sensitivity C-reactive protein; GLB—globulin; BIL—bilirubin; SBRT—stereotactic body radiation therapy; SABR—stereotactic ablative radiation therapy; TARE—transarterial radioembolization; CRA—cryoablation; HFRT—hypofractionated radiation therapy; PFS—progression-free survival; PBT—proton beam therapy; PPS—post-progression survival; PD—progressive disease; PLT—platelet; DEE-TACE—drug-eluting embolic transarterial chemoembolization; FIB—fibrinogen; GGT—gamma-glutamyltransferase; LDH—lactic dehydrogenase; PT-INR—prothrombin time–international normalized ratio; NLR—neutrophil to lymphocyte ratio; LYM—lymphocyte; FIB-4—fibrosis-4; AST—aspartate aminotransferase; PNI—prognostic nutritional index; SII—systemic immune-inflammation index; CSS—cancer-specific survival; CSPH—clinically significant portal hypertension; HBV—hepatitis B virus.
